# Automatic localization and identification of mitochondria in cellular electron cryo-tomography using faster-RCNN

**DOI:** 10.1186/s12859-019-2650-7

**Published:** 2019-03-29

**Authors:** Ran Li, Xiangrui Zeng, Stephanie E. Sigmund, Ruogu Lin, Bo Zhou, Chang Liu, Kaiwen Wang, Rui Jiang, Zachary Freyberg, Hairong Lv, Min Xu

**Affiliations:** 1 Department of Automation, Tsinghua University, Beijing, China; 2 Computational Biology Department, Carnegie Mellon University, Pittsburgh, PA USA; 3 Department of Cellular, Molecular and Biophysical Studies, Columbia University Medical Center, New York, NY USA; 4 Robotics Institute, Carnegie Mellon University, Pittsburgh, PA USA; 5 Department of Electrical and Computer Engineering, Carnegie Mellon University, Pittsburgh, PA USA; 6 Departments of Psychiatry and Cell Biology, University of Pittsburgh, Pittsburgh, PA USA

**Keywords:** Cryo-ET, Faster-RCNN, Cellular structure detection, Biomedical image analysis

## Abstract

**Background:**

Cryo-electron tomography (cryo-ET) enables the 3D visualization of cellular organization in near-native state which plays important roles in the field of structural cell biology. However, due to the low signal-to-noise ratio (SNR), large volume and high content complexity within cells, it remains difficult and time-consuming to localize and identify different components in cellular cryo-ET. To automatically localize and recognize *in situ* cellular structures of interest captured by cryo-ET, we proposed a simple yet effective automatic image analysis approach based on Faster-RCNN.

**Results:**

Our experimental results were validated using *in situ* cyro-ET-imaged mitochondria data. Our experimental results show that our algorithm can accurately localize and identify important cellular structures on both the 2D tilt images and the reconstructed 2D slices of cryo-ET. When ran on the mitochondria cryo-ET dataset, our algorithm achieved Average Precision >0.95. Moreover, our study demonstrated that our customized pre-processing steps can further improve the robustness of our model performance.

**Conclusions:**

In this paper, we proposed an automatic Cryo-ET image analysis algorithm for localization and identification of different structure of interest in cells, which is the first Faster-RCNN based method for localizing an cellular organelle in Cryo-ET images and demonstrated the high accuracy and robustness of detection and classification tasks of intracellular mitochondria. Furthermore, our approach can be easily applied to detection tasks of other cellular structures as well.

## Background

In cells, most biological processes are dominated by intricate molecular assemblies and networks. Analyzing the structural features and spatial organization of those assemblies is essential for understanding cellular functions. Recently, cellular cryo-Electron Tomography (cryo-ET) has been developed as an approach to obtain 3D visualization of cellular structures at submolecular resolution and in a close-to-native state [1]. Cryo-ET has been proven to be a powerful technique for structural biology *in situ* and has been successfully applied to the study of many important structures, including vaults [2], Integrin Linked Kinase (ILK) [3], and the nuclear pore complex (NPC) [4]. However, the systematic structural analysis of cellular components in cryo-ET images remains challenging due to several factors including low signal-to-noise ratio (SNR), limited projection range (leading to the missing wedge effect) and a crowded intracellular environment composed of complex intracellular structures.

Given the critical roles played by mitochondria within mammalian cells, and the distinctive morphology of these organelles, we chose to examine mitochondria imaged by *in situ* cryo-ET [5]. The 3D visualization of mitochondria can provide insights into mitochondrial structure and functionalities. Therefore, methodological improvements in the detection and localization of mitochondria within complex *in situ* cryo-ET datasets may significantly improve accuracy of detection of these organelles and directly impact further structural analyses.

Localization of the subcellular structures of interest can facilitate subsequent study of specific macromolecular components within the selected structures [6]. Such localization can be performed through image segmentation, which are usually performed manually or by specifically designed heuristics. Although some visualization tools have been developed to facilitate these approaches, manual segmentation in Cryo-ET images still requires large amounts of repetitive labor from researchers, and the results of which are subjective. On the other hand, automatic methods are fast and can produce consistent results. Contour-based methods like Watershed yield great results when the image complexity is low, but appear to be sensitive to noise [7]. Threshold-based methods, which usually generate a mask according to the density threshold, can be applied to foreground-background segmentation but still have difficulty in identifying different cellular components [8]. Recently, segmentation methods focusing on specific types of structures including membranes, microtubules and filaments [9–11], have drawn a lot of attention. These methods perform well on specific cellular structures, but lack generality. To date, machine learning approaches to identify intracellular structures appears to be promising. Consequently, we have developed an unsupervised segmentation method based on manually designed heuristic rules [12], and by clustering representative features [13]. Luengo et al. [14] proposed a supervised approach to classify each voxel with a trained classification model. However, both of these methods require manually designed features or rules, which might be time- and effort-consuming while having various limitations. Chen et al. developed another supervised segmentation method, taking advantage of the excellent capability of feature extraction of convolutional neural network (CNN) [15]. But in this way, a separate CNN has to be trained for each type of structural features, and the precise contours need to be manually annotated in the training data, which may not be trivial.

Our goal is to design a simple and generic method of automatic identification and localization of subcellular structures of interest within *in situ* cryo-ET images with weak annotations, which is different from existing segmentation-type methods and can greatly reduce the time and effort cost of detailed manual annotation. We aim to detect all objects of interest in an image and output corresponding bounding box with class prediction simultaneously. Region-based convolutional neural network (RCNN) [16], which generates region proposals using Selective Search, extracts features from all the proposals after normalization with CNNs, and finally feeds the features to a classifier and a regression layer simultaneously to get both classification scores and bounding box coordinates as output, lays the foundation for our goal. And its last incarnation, Faster RCNN [17], has achieved almost real-time detection with a high degree of accuracy. Faster RCNN based localization methods have been applied to biomedical imaging data such as breast mammography [18] and cellular fluorescence imaging [19].

In this work, we proposed an automatic identification and localization method based on Faster-RCNN, which is the first Faster-RCNN based method for localizing an cellular organelle in Cryo-ET images. Our algorithm is trained and validated on 2D projection images of a cryo-ET tomogram for localization and classification tasks of mitochondira. Our experimental results show that our algorithm is able to robustly predict the object’s bounding box with classification scores. Moreover, we extended our study to 3D tomogram slices and achieved accurate and robust performance.

## Method

**Fig. 1:**
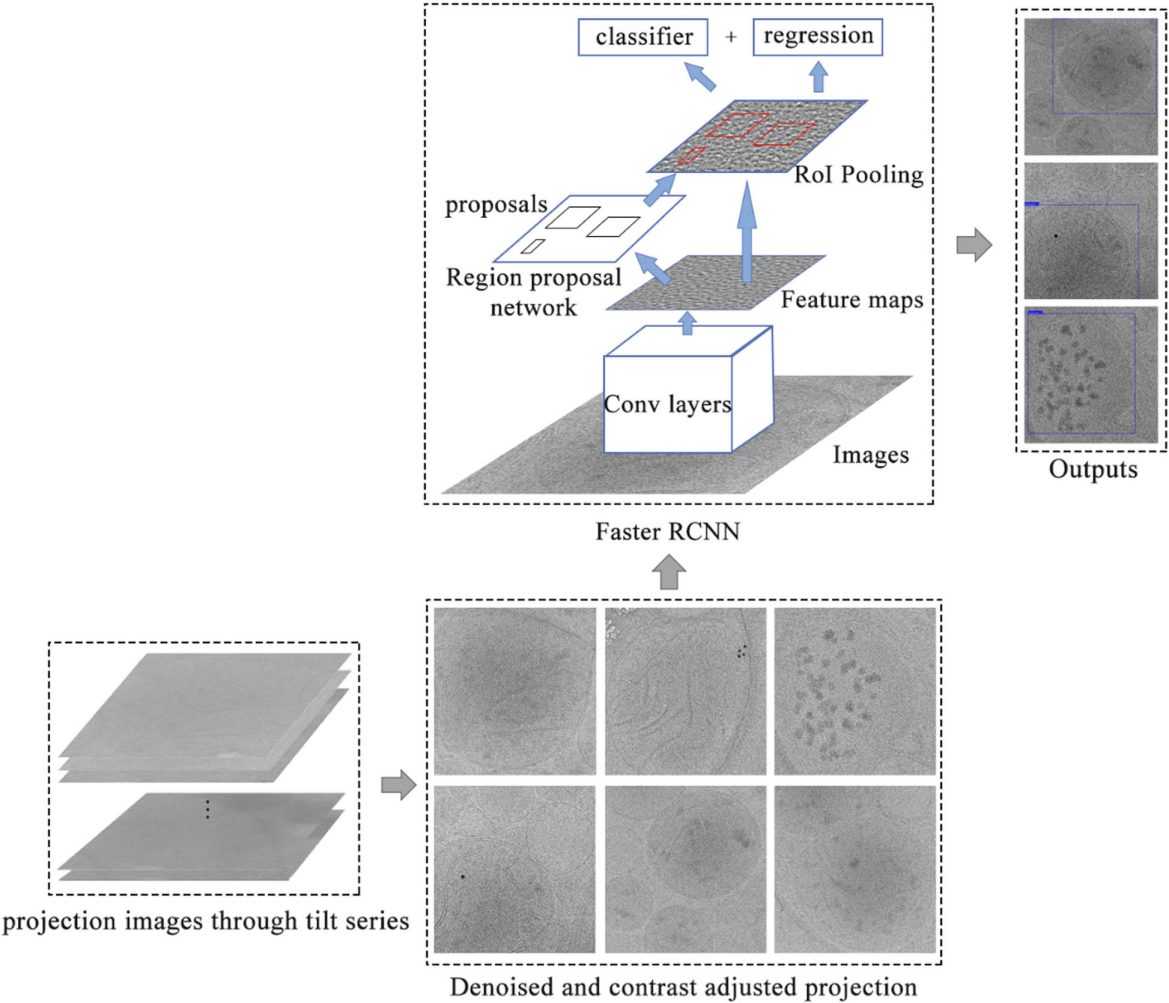
Flowchart of our Faster-RCNN model. The denoised input image is fed into Conv layers to generate the feature map. Then, region proposal network proposes potential regions that contain object of interest. The proposal regions are passed to 1) classifier for classification, 2) regressor for refine the bounding box location

Our mitochondria identification and localization method is comprised of two main parts: (1) pre-processing to improve the quality of samples, and (2) object detection using Faster-RCNN. The input of our system is 2D projection images of a tomogram, and the output includes coordinates of the bounding boxes of object of interest, the class of each object and the probability of the classification. A flowchart of our method is shown in Fig. 1. In this section, we willdescribe each part of our system in details.

### Preprocessing

Since biological samples are sensitive to radiation damage, only low-dose electrons can be used for electron microscopy imaging [6]. Compared to normal images, electron tomography images are usually noisier and have lower contrast. To make the images suitable for subsequent processing, we first perform noise reduction and contrast enhancement. To reduce noise, considering the edge features are often important for subcellular structures, we chose Bilateral Filtering [20], a nonlinear filtering method that preserves the original edges as much as possible. Bilateral Filtering considers the effects of both spatial distance and gray scale distance, and can be implemented by combining two Gaussian Filters. To improve local contrast and the definition of details, we use Histogram Equalization, which can also balance the brightness of different images.

### Object detection in 2D images

The main idea of our method is based on Faster RCNN [17], in which the four modules of feature extraction, proposal generation, RoI Pooling, classification and regression are organically combined to form an end-to-end object detection system.

**Fig. 2:**
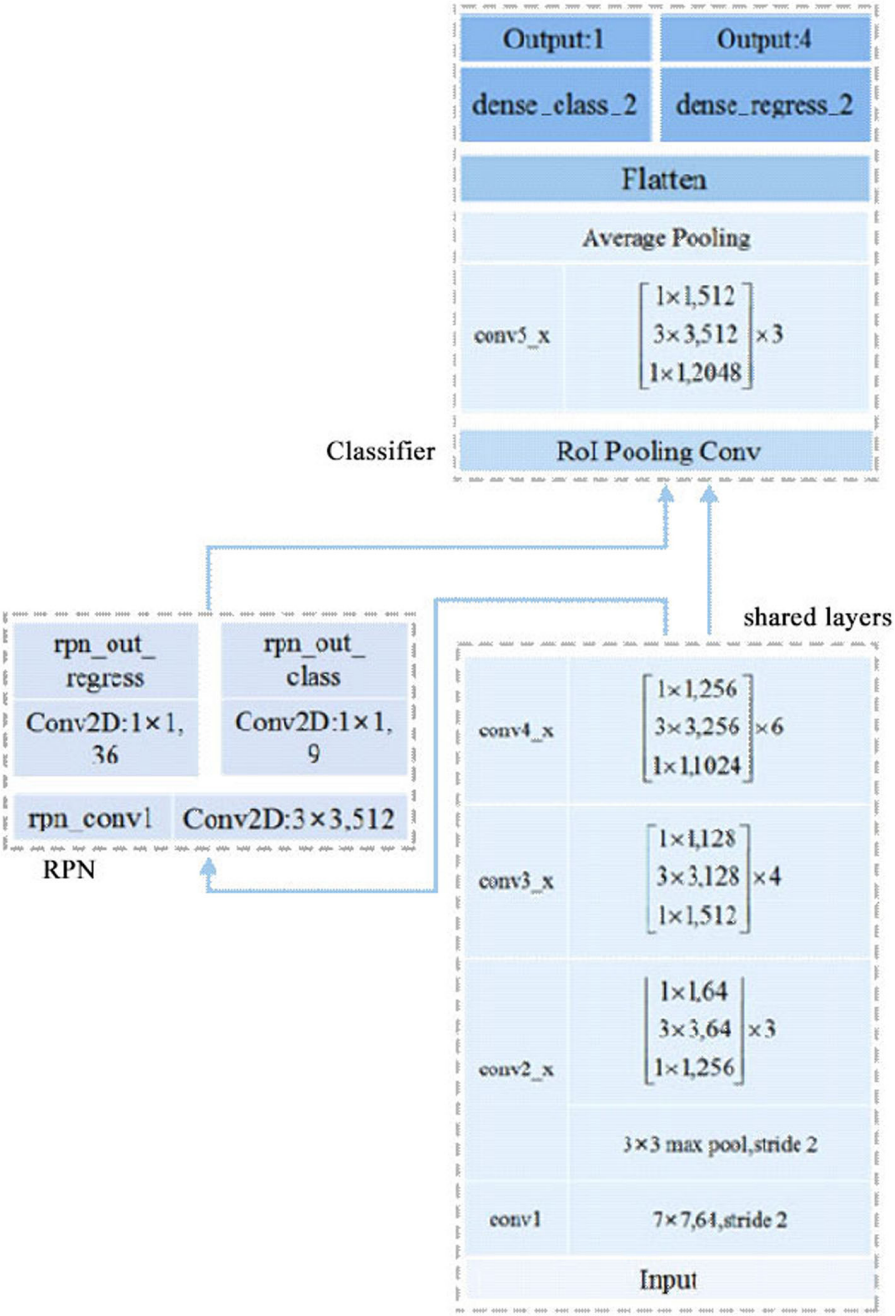
Detailed Architecture of the Faster-RCNN model. The basic feature extraction network Resnet-50 is split into two parts in our model: 1) layers conv1 to conv4_x is used for extraction of shared features (in the shared layers), 2) layer conv5_x and upper layers further extracts features of proposals for the final classification and regression (in the classifier). And the RPN implemented with three convolutional layers generates proposals from the shared feature map

Feature extraction is the first step of our method. The input of the deep convolutional neural network is the image *I*, and the output is the extracted feature map. These features will be shared by subsequent modules. The basic feature extraction network in our model, Resnet-50, is based on [21]. He et al. proposed this deep residual learning method in 2015 to make the deeper network train properly. The architecture of our network is shown in Fig. 2. The original Resnet-50 network is split into two parts in our model: part one including layers conv1 to conv4_x is used for extraction of shared features, and part two including layer conv5_x and upper layers further extracts features of proposals for the final classification and regression. The implementation of the model refers to the work of Yann Henon in 2017 [22].

The feature extraction network is followed by a region proposal network (RPN). A window of size *n*×*n* slides onto the feature map, and at each location it stays the features in the window are mapped to a low-dimensional vector, which will be used for object-background classification and proposal regression. At the same time, *k* region proposals centered on the sliding window in the original image are extracted according to *k* anchors, which are rectangular boxes of different shapes and sizes. Moreover, for each proposal, two probabilities for the classification and four parameters for the regression will be achieved, composing the final 6*k* outputs of the classification layer and the regression layer. The sliding window, classification layer and regression layer are all implemented using convolutional neural networks. In practice, we chose *k*=9 with 3 scales of 128^2^, 256^2^, and 512^2^ pixels and 3 aspect ratios of 1:1, 1:2, and 2:1 as the default in [17]. And non-maximum suppression(NMS) was adopted with the IoU threshold at 0.7, while the maximum number of proposals produced by the RPN was 300.

Features of different scales are then integrated into feature maps of the same size (7×7 in our experiment) via RoI pooling layer, so that the features can be used in final fully connected classification and regression layers. For a region proposal of any size, like *h*×*w*, it will be divided into a fixed number, like *H*×*W*, of windows of size *h*/*H*×*w*/*W*. Then max pooling will be performed and a fixed-size (*H*×*W*) feature map will be obtained with the maximum of each window.

To train the whole model end-to-end, a multi-task loss function is proposed as follows [17].

1 $$ L\left(p,u,t^{u},v\right)=L_{cls}(p,u)+\lambda[u\geq 1 ]L_{loc}\left(t^{u},v\right)  $$

Where *u* is the ground truth label of the proposal, and *v*=(*v*_*x*_,*v*_*y*_,*v*_*w*_,*v*_*h*_) represents the regression offset between the proposal and the ground truth.The output of the classification layer, *p*=(*p*_0_,*p*_1_,...,*p*_*K*_), represents the probabilities of the proposal belonging to each one of the *K*+1 classes and $t^{u}=\left (t_{x}^{u},t_{y}^{u},t_{w}^{u},t_{h}^{u}\right)$ represents the predicted regression offset for a proposal with label *u*. The loss function of the classification task is defined as:

2 $$ L_{cls}(p,u)=-\log p_{u}.  $$

And the loss function of the regression is a robust L1 loss as follows:

3 $$ L_{loc}\left(t^{u},v\right)=\sum_{i\in {x,y,w,h}}smooth_{L1}\left(t_{i}^{u}-v_{i}\right).  $$

Where

4 $$ smooth_{L}1\left(x \right)=\left\{ \begin{array}{lr} 0.5x^{2}, \: \: \: \: \: if \, \|x\|<1 & \\ \|x\|-0.5, \: \: \: \: \: otherwise & \end{array} \right.  $$

The hyperparameter *λ* is used to control the balance between the two losses and is set to *λ*=1 in our experiment. Similarly, the loss function of the RPN during training is also defined in this form. In the training process, the RPN with the shared layers is trained first and then the classifier is trained using proposals generated by the RPN, with the initial weights for both networks given by a pretrained model on ImageNet [17, 23].

## Results

### Dataset and evaluation metrics

**Data Acquisition**: Tissue Culture: Rat INS-1E cells (gift of P. Maechler, Université de Genève) were cultured in RPMI 1640 medium supplemented with 2 mM L-glutamine (Life Technologies, Grand Island, NY), 5% heat-inactivated fetal bovine serum, 10 mM HEPES, 100 units/mL penicillin, 100 *μ*g/mL streptomycin, 1 mM sodium pyruvate, and 50 *μ*M b-Mercaptoethanol as described earlier (insert reference: PMID: 14592952).

EM Grid Preparation: For cryo-ET imaging, INS-1E cells were plated onto either fibronectin-coated 200 mesh gold R2/1 Quantifoil grids or 200 mesh gold R2/2 London finder Quantifoil grids (Quantifoil Micro Tools GmbH, Jena, Germany) at a density of 2×10^5^ cells/mL. Following 48 h incubation under conventional culture conditions in complete RPMI 1640 medium, grids were removed directly from culture medium and immediately plunge frozen in liquid ethane using a Vitrobot Mark IV (Thermo Fisher FEI, Hillsboro, OR).

Cryo-Electron Tomography: Tomographic tilt series for INS-1E cells were recorded on a FEI Polara F30 electron microscope (Thermo Fisher FEI) at 300kV with a tilt range of ±60° in 1.5° increments using the Gatan K2 Summit direct detector (Gatan, Inc.) in super-resolution mode at 2X binned to 2.6 Å/pixel; tilt series were acquired via SerialEM.

**Datasets**: We collected 9 cryo-ET tomograms (786 2D slices) contains mitochondria. 482 out of the 786 slices were selected and annotated manually via LabelImg [24]. Then, the 2D slices were randomly divided into training and testing set with a ratio of 5:1. Details of our dataset are shown in Table 1.

**Table 1:** Cryo-ET dataset properties

Tomogram basename	Image size	All slices	Used slices
Unstim_20k_mito1	3708×3838	101	75
Unstim_20k_mito2	3708×3838	89	44
CTL_Fibro_mito1	3708×3838	82	36
M2236_Fibro_mito2	3708×3838	90	46
M2236_turemito3	3708×3838	86	39
CHX + Glucose Stimulation A2	3708×3838	53	51
HighGluc_Mito1	3708×3838	101	71
HighGluc_Mito2	3708×3838	101	69
INS_21_g3_t10	3708×3838	81	51
Total		786	482

**Metrics**: To evaluate the performance of our model, we mainly use two metrics from common object detection and segmentation evaluation: AP (average precision) and *F*_1_ *score*. The definitions are as follows:

5 $$ AP=\int_{0}^{1} P(R)\,d(R)  $$

6 $$ F_{1} \ score=\frac{2P \times R}{P+R}  $$

where *P* represents precision, which indicates the ratio of the true positives to all predicted positives; *R* represents recall, which indicates the ratio of the true positives to all true elements. Neither precision nor recall alone is sufficient to fully evaluate the prediction performance. Therefore, the F1 score defined by the weighted harmonic mean of precision and recall is commonly used in the case where both of them need to be high enough. And AP, equivalent to the area under the precision-recall curve, may provide an overall evaluation of the model’s performance at different precision/recall rates. As an object detection problem, the correctness of each sample prediction is not only related to classification, but also related to localization. The accuracy of localization is evaluated by (Intersection over Union), which is defined as:

7 $$ IoU=\frac{S_{P} \cap S_{G}}{S_{P} \cup S_{G}}  $$

where *S*_*P*_ is the predicted bounding box and *S*_*G*_ represents the ground truth, and IoU measures the degree of coincidence. In our experiments, different IoU thresholds(0.5, 0.6, 0.7, 0.8, and 0.9) are set, and those samples with mitochondria prediction labels and IoUs higher than the specific threshold are considered. The higher the IoU threshold, the higher the accuracy requirements for localization. Thus we can see the difference in the detection accuracy under different localization accuracy requirements, and judge the localization performance of our model. The precision, recall, F1 score and AP in our experiment are calculated.

### Data preprocessing and model training

**Fig. 3:**
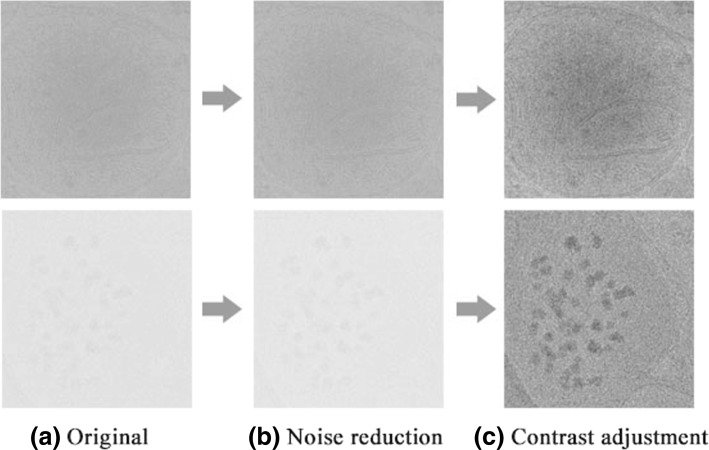
**a** Original 2D projection images, **b** Images after noise reduction (Bilateral Filtering with *σ*_*r*_=1.2 and *σ*_*d*_=100), **c** Images after noise reduction and contrast adjustment

**Fig. 4:**
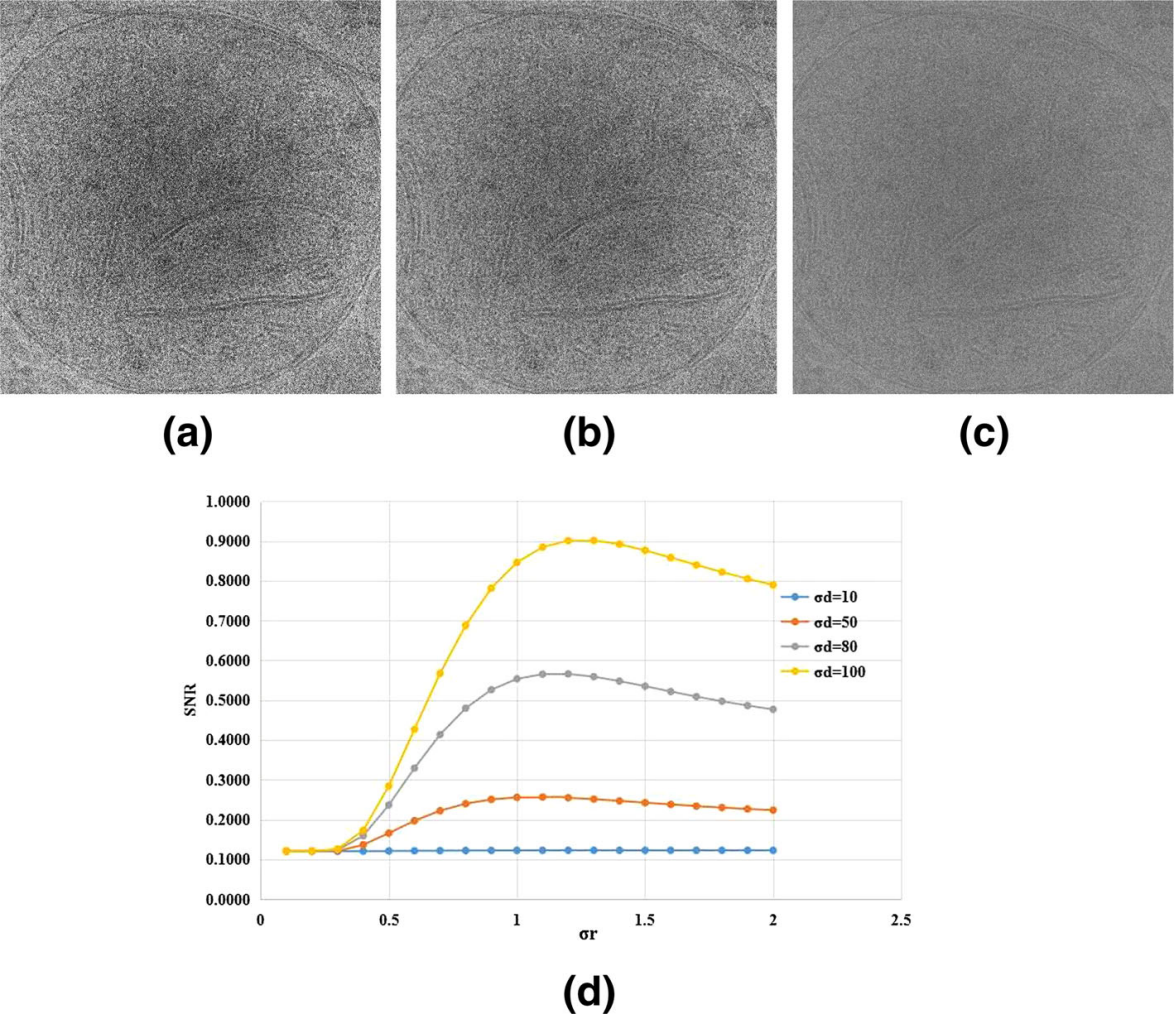
**a** Bilateral Filter + Histogram Equalization, **b** Gaussian Filter + Histogram Equalization, **c** Histogram Equalization + Bilateral Filter **d** SNR with different *σ*_*d*_ and *σ*_*r*_

The 2D projection images we acquired from the original tomograms have low SNR and contrast which interferes with subsequent identification and segmentation of intracellular features. Thus, the images are first denoised via a bilateral filter with *σ*_*r*_=1.2 and *σ*_*d*_=100, suppressing noise and retaining the original edge features as much as possible. This is followed by enhancement of contrast via histogram equalization which improves in the resolution of previously indistinguishable details. Figure 3 shows an example of two images before and after preprocessing. The preprocessing methods and parameters in our method were finally determined based on the single-image SNR estimated according to [25], gray-scale distribution histograms and visual effect of the image. Figure 4 shows SNR of the same image with different *σ*_*d*_ and *σ*_*r*_ and the performance of different preprocessing schemes. We found that performing histogram equalization first will increase the noise in the original image, and the contrast will be reduced again after filtering, failing to achieve the desired effect. Furthermore, we found that Gaussian filtering used for noise reduction cannot preserve the edge as well as Bilateral filtering.

All the models in our experiments were trained and tested using Keras [26] with Tensorflow [27] as the back-end, using optimizer Adam (Adaptive Moment Estimation) [28] with *β*_1_=0.9,*β*_2_=0.999 and learning rate of 1×10^−5^ for both RPN and the classifier. The 482 annotated slices were randomly split into a training set of 402 slices and a test set of 80 slices according to a ratio of 5:1. The model would be saved only if the loss after one epoch is less than the best loss before.

### Prediction performance

**Fig. 5:**
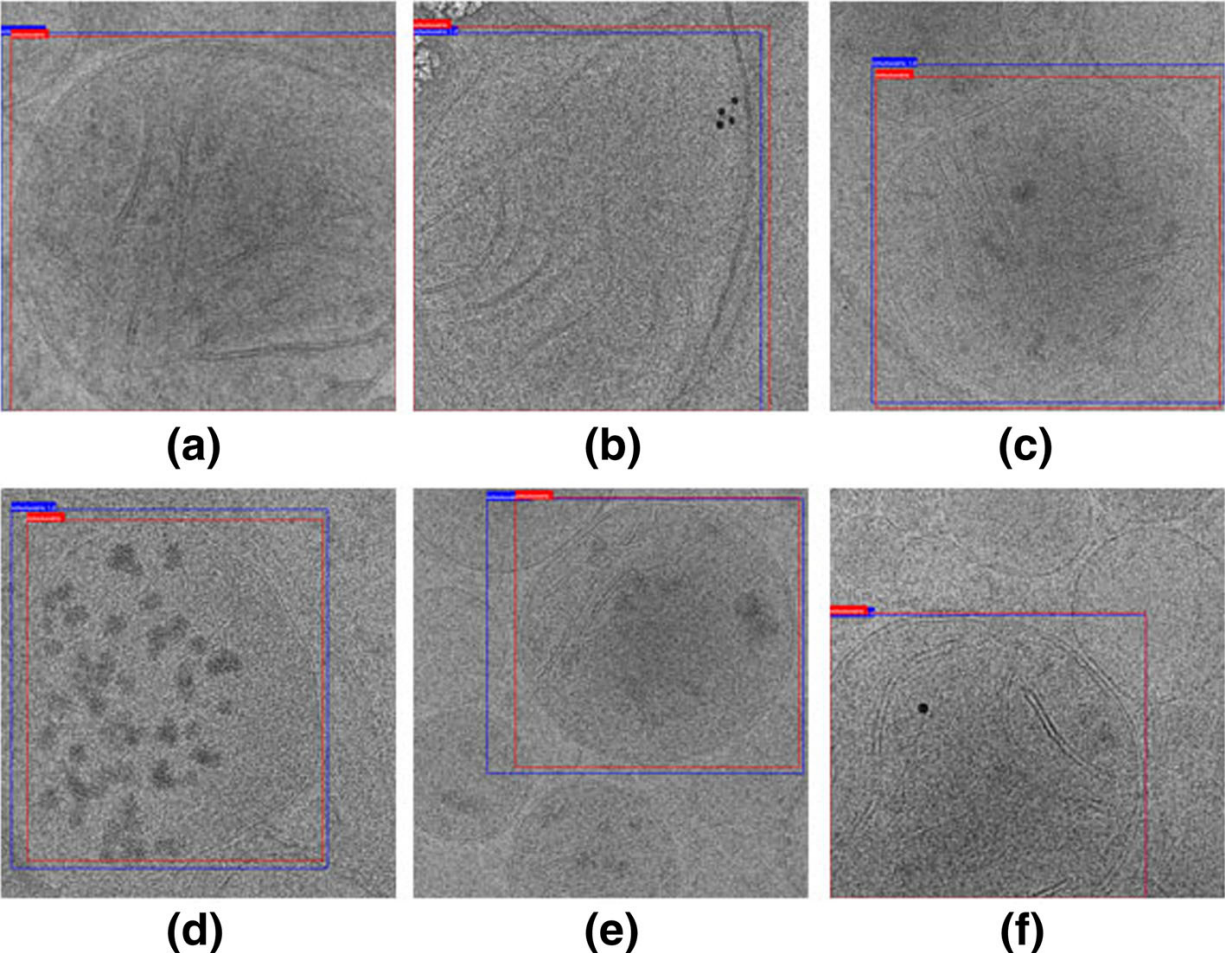
Examples of detection results: the red boxes are ground truth, and the blue ones are the predicted bounding boxes. Data source: **a** Tomogram: Unstim_20k_mito1 (projection image 63), **b** Tomogram: Unstim_20k_mito2 (projection image 49), **c** Tomogram: HighGluc_Mito2 (projection image 47), **d** Tomogram: CTL_Fibro_mito1 (projection image 44), **e** Tomogram: HighGluc_Mito1 (projection image 48), **f** Tomogram: CHX + Glucose Stimulation A2 (projection image 13)

**Fig. 6:**
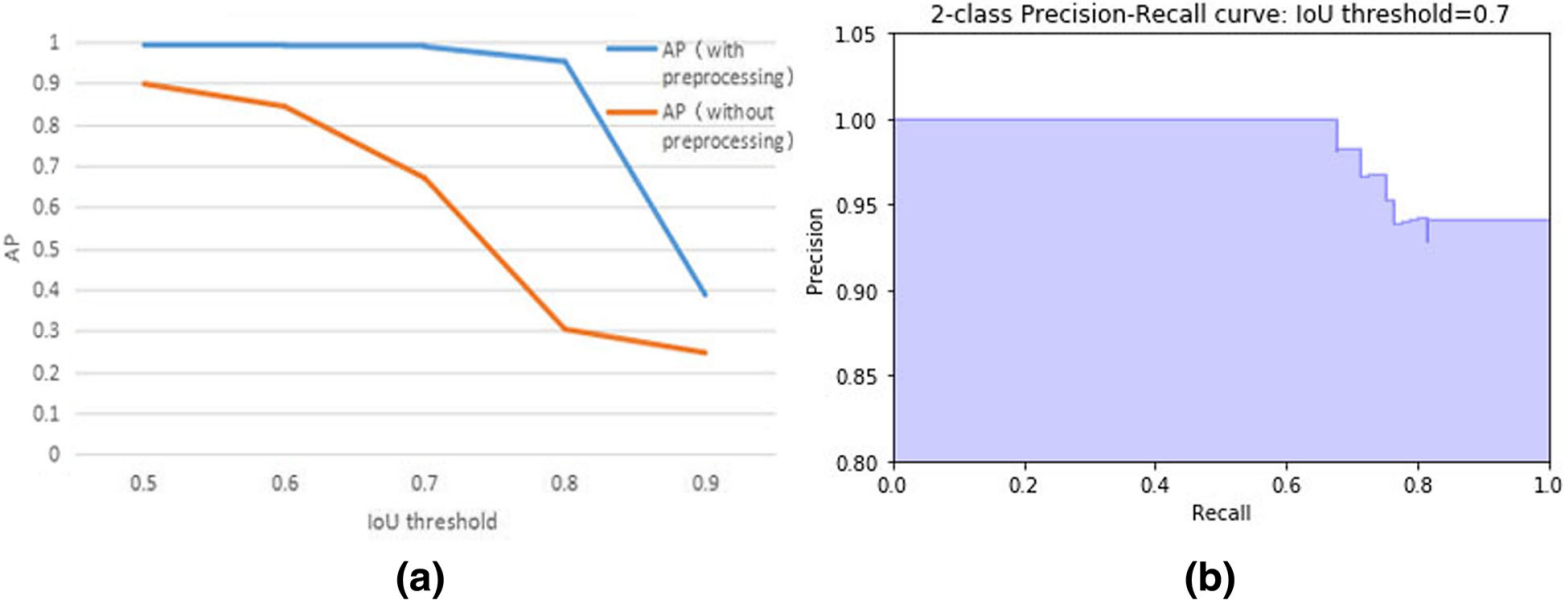
Prediction performance: **a** AP with different IoU threshold, **b** Precision-Recall curve with IoU threshold=0.7

We trained the model on the training set and tested it on the test set. Figures 5 and 6 show the test results visually and quantitatively. In addition to the bounding box, our model also gives the most likely category of the object and the probability of it belonging to that category. In Fig. 5, the red bounding box is the manually annotated ground truth, and the blue box is predicted by the model. We notice that the predicted results and the ground truth are highly coincident, and even the regions that cannot be completely overlapped basically contain the entire mitochondria, which means that our system can achieve the goal of automatic identification and localization of mitochondria quite successfully. The area where the mitochondria is located can be separated from the outside by the bounding box, so as to eliminate the influence of the surrounding environment as much as possible, making it possible to analyze the internal structures in more detail.

In Fig. 6, we plotted the precision-recall curve and calculated the APs at different IoU thresholds to measure the detection performance. We noticed that when the IoU threshold is set to 0.7 and below, the AP is close to 1, which means that almost all samples were correctly predicted,indicating that our system can successfully identify the mitochondria in the picture. However, when the IoU threshold is increased to 0.9, the AP drops sharply to around 0.4, which indicates that our system still has some deficiencies in the accuracy of localization. The overlap between the predicted area and the ground truth area can be further improved, which can be an important aspect of our future work. The precision-recall curve for IoU thresholds of 0.7 is also given in Fig. 6. When the IoU threshold is 0.7, all positive samples can be correctly predicted while the precision requirement is not higher than 0.9, that is, all mitochondria can be found in that condition; even with a precision of 1, which means all samples predicted to be positive must be correct, 70% of the mitochondria can still be detected.

In addition, we compared the effect of preprocessing on the prediction results. It is noted that no matter how the IoU threshold is set, the AP value of the model without preprocessing is significantly lower than that of the model containing the preprocessing, which again shows that preprocessing is a necessary step for the overall system. Especially when the IoU threshold is 0.8, the system with or without preprocessing shows a great difference in the average precision of prediction, which indicates that the main contribution of preprocessing to the system is to further improve the accuracy of localization. For the model that does not include preprocessing, the predicted bounding box that has an IoU no less than 0.8 with ground truth is quite rare, and the average precision calculated in this situation is only 0.3. After the preprocessing step, it becomes common that IoU of the predicted bounding box and the ground truth reaches 0.8, resulting in an increase of the average precision to 0.95 and higher.

### Source of error

**Fig. 7:**
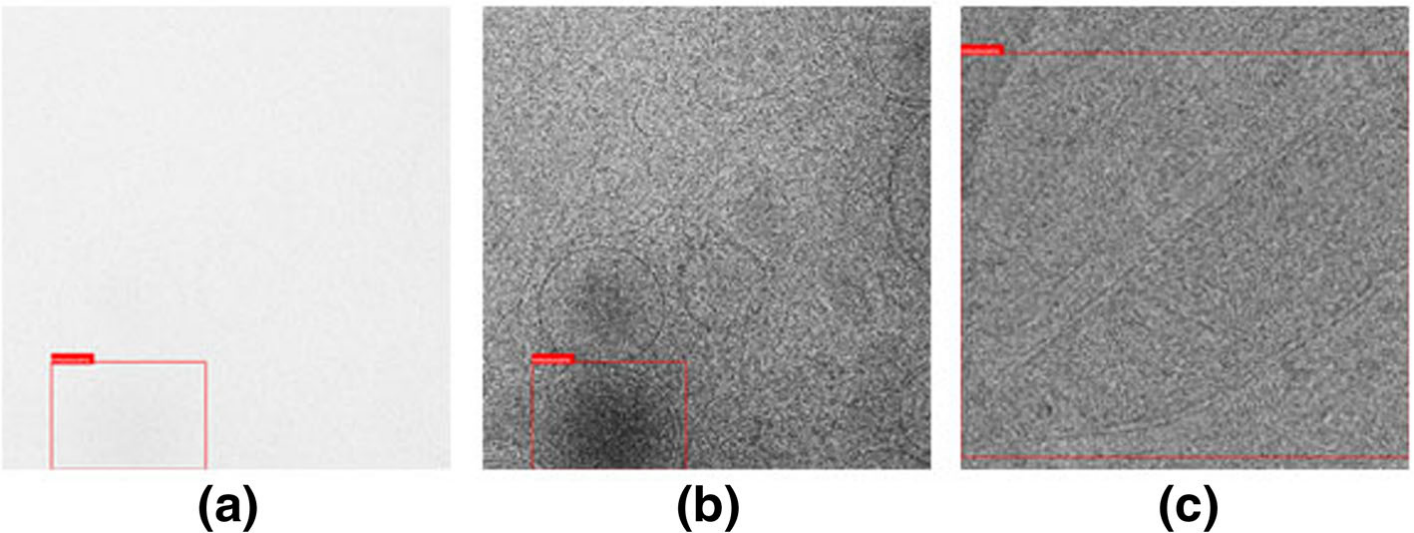
An example of projection images from tomogram INS_21_g3_t10 (in which the mitochondria is hard to detect): **a** Original image, **b** Image after noise reduction and contrast adjustment, **c** Projection image from M2236_Fibro_mito1

In order to further analyze the performance of our method, we separately analyzed the prediction results of the system on 9 different *in situ* cryo-ET tomograms (Table 2), and studied the impact of different factors including the quality of the original image, the intactness of the mitochondria etc. The *F*_1_ *score* and AP remain calculated at an IoU threshold of 0.7. In most tomograms, our systems show high accuracy, consistent with the overall results. However, we also found that in INS_21_g3_t10, our system could not accurately detect mitochondria. Therefore, we analyzed the projected image from INS_21_g3_t10 (Fig. 7). We noticed that in all the 2D projection images from that tomogram, the mitochondria included are too small and the structure appeared incomplete, especially the internal structure, which is basically submerged in noise and hard to identify. Even after noise reduction and contrast adjustment, the details of the mitochondria in the image are still too blurred, causing strong interference in the extraction of features. We also calculated the SNR of the two-dimensional projection images in INS_21_g3_t10, which is approximately 0.06 on average. For reference, the SNR of the original projection image from Unstim_20k_mito1 we analyzed in Fig. 4 is 0.12, which is significantly higher than the images in INS_21_g3_t10. It is also worth noting that in Unstim_20k_mito1, the subject of the projection images is the mitochondria we need to detect, while in INS_21_g3_t10, the mitochondria only occupy a very small part of the image. As a result, other components of the image are calculated as signal which may be not that useful for our detection task, making the ratio of effective information to noise even lower than 0.06. This may explain why the detection performance of it is particularly unsatisfactory.

**Table 2:** Prediction results on different tomograms

Tomogram basename	*F*_1_ *s* *c* *o* *r* *e*	AP	mIoU	Incomplete mitochondria
Unstim_20k_mito1	0.91	0.98	0.826	YES
Unstim_20k_mito2	1	1	0.864	NO
CTL_Fibro_mito1	0.97	0.99	0.843	NO
M2236_Fibro_mito2	0.96	0.99	0.887	YES
M2236_turemito3	0.91	0.97	0.783	NO
CHX + Glucose Stimulation A2	0.94	1	0.75	YES
HighGluc_Mito1	0.97	0.99	0.843	NO
HighGluc_Mito2	0.97	0.96	0.837	NO
INS_21_g3_t10	0	0	0	YES

The *F*_1_ *score* and AP are calculated at an IoU threshold of 0.7

In order to better study the influence of different tomograms on the accuracy of localization, mean Intersection over Union (mIoU) is calculated for each tomogram. It can be noted that, on average, mIoU is higher in the tomograms that contain complete mitochondria, that is, the localization accuracy is higher, although the highest mIoU comes from a tomogram containing incomplete mitochondria. We analyzed the characteristics of this tomogram and found that it is the only one where mitochondria do not appear circular or nearly circular, but instead possess a slanted strip shape (also shown in Fig. 7). Therefore, when the mitochondrion is marked with a rectangular box, the box occupies a larger area and contains more non-mitochondrial regions, which may make the prediction results more easily coincide with the ground truth. Therefore, in general, we can still conclude that complete mitochondria are more easily localized accurately. This is also in consistent with our intuition that the complete mitochondria have a complete outline of a bilayer membrane that approximates a circular shape, which provides a powerful reference for determining its specific boundaries. In fact, the tomogram with best results on the *F*_1_ *score* and AP also contains intact mitochondria. Therefore, the integrity of mitochondria has a certain impact on the detection results of the system.

### Prediction on tomogram slices

The ultimate goal is to detect mitonchondria in 3D tomograms. The model trained on 2D projection images can be directly applied to tomogram slices to generate the output. Like projection images, the slices were first preprocessed through Bilateral filtering and histogram equalization with the same parameters, and then tested by the Faster-RCNN model. The whole model is applied to the tomogram slice by slice and the output includes all the bounding boxes of mitochondria in the slice with a classification score for each box. And it only takes a few seconds for each slice when tested on CPUs.

**Fig. 8:**
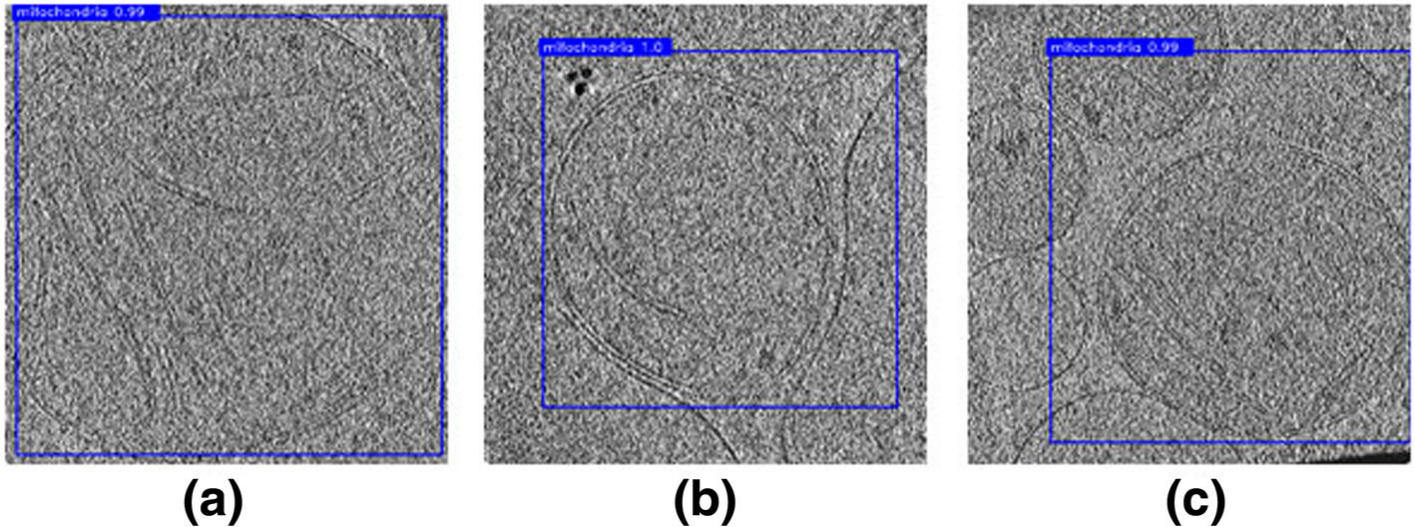
Detection results on slices of reconstructed tomograms. Data source: **a** Tomogram: Unstim_20k_mito_1 (slice 26), **b** Tomogram: M2236_truemito3 (slice 97), **c** Tomogram: HighGluc_Mito1 (slice 58)

As shown in Fig. 8, the mitochondria in tomogram slices can be successfully identified and localized, while the accuracy of localization may be slightly reduced due to higher noise, as compared to 2D projection images. Therefore, it is only necessary to perform annotation and training on the 2D projection images, which can greatly reduce the computational costs, and we can detect mitochondria in 3D tomograms with a tolerable error. And the probability of expanding to different organelles is still retained even in the case of 3D.

## Conclusion

In this paper, we proposed an automatic Cryo-ET image analysis algorithm for localization and identification of different structure of interest in cells. To best to our knowledge, this is the first work to applied Faster-RCNN model to Cryo-ET data, which demonstrated the high accuracy (*AP*>0.95 and *IoU*>0.7) and robustness of detection and classification tasks of intracellular mitochondria. Furthermore, our algorithm can be generalized to detect multiple cellular components using the same Faster-RCNN model, if annotations of multiple classes of cellular component were provided. For future work, we will further improve the accuracy of localization by collecting more data and we will explore the effects of different network structures to enhance the model.

## Abbreviations

Adam: Adaptive moment estimation
AP: Average precision
CNN: Convolutional neural network
cryo-ET: Cryo-electron tomography
ILK: Integrin linked kinase
IoU: Intersection over union
mIoU: Mean intersection over union NMS: Non-maximum suppression
NPC: Nuclear pore complex
SNR: Signal-to-noise ratio
RCNN: Region-based convolutional neural network
RPN: Region proposal network
